# Dissecting cardiovascular disease-associated noncoding genetic variants using human iPSC models

**DOI:** 10.1016/j.stemcr.2025.102467

**Published:** 2025-03-20

**Authors:** Saif F. Dababneh, Hosna Babini, Verónica Jiménez-Sábado, Sheila S. Teves, Kyoung-Han Kim, Glen F. Tibbits

**Affiliations:** 1 Department of Cellular and Physiological Sciences, University of British Columbia, Vancouver, BC V6T 1Z3, Canada; 2 Cellular and Regenerative Medicine Centre, BC Children’s Hospital Research Institute, 938 West 28th Avenue, Vancouver, BC V5Z 4H4, Canada; 3 Departments of Molecular Biology and Biochemistry / Biomedical Physiology and Kinesiology, Simon Fraser University, Burnaby, BC V5A 1S6, Canada; 4 Department of Biochemistry and Molecular Biology, Life Sciences Institute, University of British Columbia, Vancouver, BC V6T 1Z3, Canada; 5 Department of Cellular and Molecular Medicine, Faculty of Medicine, University of Ottawa, Ottawa, ON K1H 8M5, Canada; 6 University of Ottawa Heart Institute, Ottawa, ON K1Y 4W7, Canada; 7 School of Biomedical Engineering, University of British Columbia, Vancouver, BC V6T 2B9, Canada

**Keywords:** noncoding variants, regulatory elements, iPSCs, cardiomyocytes, epigenetics, cardiovascular disease, genetics

## Abstract

Advancements in genomics have revealed hundreds of loci associated with cardiovascular diseases, highlighting the role genetic variants play in disease pathogenesis. Notably, most variants lie within noncoding genomic regions that modulate transcription factor binding, chromatin accessibility, and thereby the expression levels and cell type specificity of gene transcripts. Human induced pluripotent stem cell-derived cardiomyocytes (hiPSC-CMs) have emerged as a powerful tool to delineate the pathogenicity of such variants and elucidate the underlying transcriptional mechanisms. Our review discusses the basics of noncoding variant-mediated pathogenesis, the methodologies utilized, and how hiPSC-based heart models can be leveraged to dissect the mechanisms of noncoding variants.

## Teaser

In this review, we discuss the basics of noncoding variant-mediated pathogenesis, the methodologies utilized in delineating such mechanisms, and how hiPSC-CMs can be leveraged to dissect the pathogenic mechanisms of causal noncoding variants. We also highlight the current strengths and limitations of hiPSC-CMs and discuss recent advancements in the field, such as 3D cardiac organoids, cell villages, and chimeroids, which will further propel the study of human variation. Finally, we thoroughly summarize epigenetic datasets generated in hiPSC-based heart models, which we believe will greatly benefit the scientific community.

## Introduction

Cardiovascular disease is the leading cause of mortality worldwide, and genetic susceptibility plays a critical role in the risk of cardiovascular disease but remains less well understood (Vaduganathan et al., 2022; Walsh et al., 2023). Advanced techniques, such as next-generation sequencing and genome-wide association studies (GWASs), have identified numerous genetic susceptibility loci, generating an immense amount of genetic data that require in-depth investigation. Initial attempts at examining these loci have centered on genetic variants that occur within protein-coding sequences, which directly affect protein sequence and function. However, the majority of identified genetic variants are located within noncoding genomic regions, which do not directly alter the exome (i.e., protein-coding sequences) (Yu et al., 2024). Instead, these noncoding variants are often found within regulatory DNA elements, including promoters, enhancers, and silencers, and in regions critical to the genome’s three-dimensional (3D) structure. These noncoding regions are critical for the precise spatiotemporal coordination of transcriptional processes underpinning cardiac development and function (Akerberg and Pu, 2020).

However, the complexity of noncoding regions poses a significant challenge in discerning which variants are pathogenic and how they contribute to dysfunction. This complexity also serves as a major barrier to routine clinical implementation for disease prediction and prognostication (Ellingford et al., 2022). Understanding the impact of noncoding variants requires investigating the transcriptome and epigenome, which renders the process technically and financially burdensome. Moreover, the impact of most genetic variants, especially noncoding variants, may also depend on an individual’s genomic background. In some cases, the transcriptional alterations conferred by a noncoding variant may be counterbalanced by those from another (Zhang and Lupski, 2015). This complexity highlights the importance of model systems that enable the investigation of noncoding variants within the context of human genetic diversity.

In recent years, human induced pluripotent stem cell-derived cardiomyocytes (hiPSC-CMs) have been rapidly adopted as a model system to investigate the impact of genetic variation on cardiovascular diseases in the context of human cardiomyocytes (CMs). This approach has particularly flourished in the areas of cardiotoxicity, inherited cardiac disorders (e.g., arrhythmias and cardiomyopathies), and pharmacogenomics (Magdy and Burridge, 2021). Since hiPSC-CMs are derived from unique individuals and can serve as a largely scalable source of human CMs, they have become instrumental in the mechanistic dissection of noncoding variants and their roles in disease. These efforts have generated invaluable transcriptomic and epigenomic datasets that benefit the scientific community at large (Caudal et al., 2024). In combination with *in vivo* models, hiPSC-CMs offer a powerful model system for understanding the effects of noncoding genetic variants at both cellular and whole organ levels and for developing prediction tools that account for genome-wide risk.

In this review, we discuss the basics of noncoding variant-mediated pathogenesis, the methodologies utilized in delineating such mechanisms, and how hiPSC-CMs can be leveraged to dissect the pathogenic mechanisms of causal noncoding variants. We also highlight the current strengths and limitations of hiPSC-CMs and discuss recent advancements in the field, including cardiac organoids, cell villages, and chimeroids, which will further propel the study of human variation.

## Primer on gene regulation and mechanisms underlying noncoding variants

As the first step in gene expression, transcription is an essential and highly regulated process, which begins when the transcriptional machinery is loaded onto gene promoters. Enhancers are conserved DNA elements that provide precise spatiotemporal regulation, often functioning over long genomic distances, by containing binding sites for specific transcription factors (TFs) that, in turn, recruit coactivators that bridge the enhancers to the promoter region (Andersson and Sandelin, 2020). A critical mechanism through which enhancers exert their effect is chromatin looping, where the DNA physically loops to bring the enhancer in proximity to the gene’s promoter. This spatial organization allows for direct interactions between the enhancer-bound TFs and the promoter, facilitating efficient transcription initiation. Chromatin loops that help regulate gene expression occur throughout the genome and are often anchored by DNA-binding factors such as CTCF and cohesin, whose sequence-binding motifs determine the 3D organization of chromatin (Popay and Dixon, 2022). In addition, histone modifications add another layer of regulation by dictating the accessibility of DNA through the modulation of chromatin packing in the form of euchromatin (accessible) or heterochromatin (inaccessible) (Bannister and Kouzarides, 2011).

**Figure 1:**
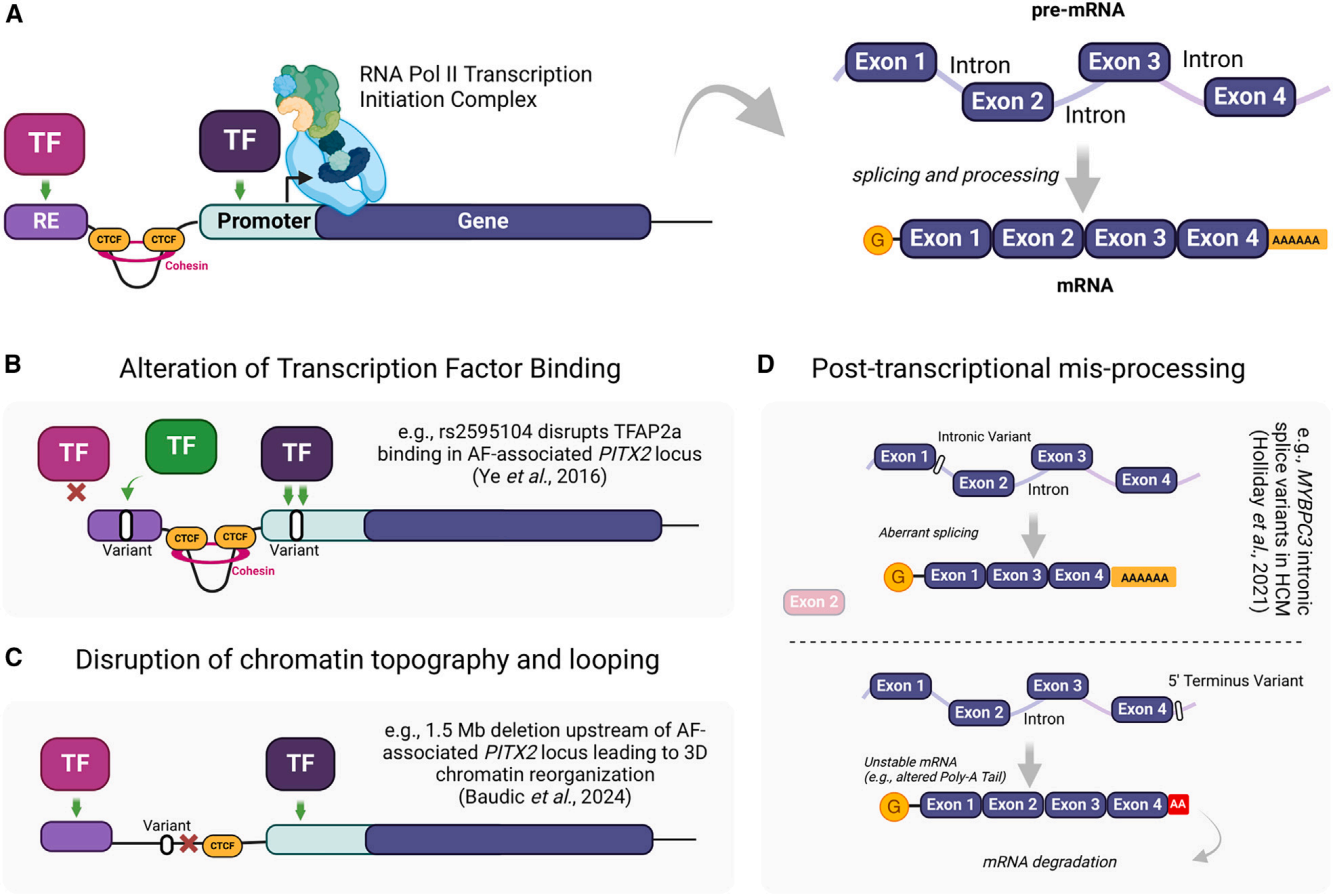
Molecular mechanisms of noncoding variants Graphical summary demonstrating the main mechanisms of noncoding variant-mediated dysfunction. (A) Key regulatory features of transcription and post-transcriptional processes. (B) Noncoding variants within regulatory elements can disrupt transcription factor binding by reducing binding affinity (light purple TF), strengthening binding affinity (dark purple TF), and/or creating *de novo* binding sites for other TFs (green TF). (C) Noncoding variants in regulatory elements required for chromatin looping (e.g., CTCF-binding site) can alter chromatin topography. (D) Noncoding variants in intronic regions can result in aberrant splicing (top) or affect mRNA stability by disrupting the process of poly-adenylation as an example (bottom). A cardiac example is highlighted in text in (B)–(D). TF, transcription factor; RE, regulatory element; Pol, polymerase; AF, atrial fibrillation; HCM, hypertrophic cardiomyopathy. Created with BioRender.

Noncoding variants can contribute to disease by potentially disrupting one of several transcriptional or post-transcriptional processes (French and Edwards, 2020). Some of the main pathways include disrupting binding sites for transcriptional regulators, altering the 3D conformation of chromatin topography, or affecting post-transcriptional processes such as alternative splicing or mRNA stability, as summarized in Figure 1.

Most noncoding variants within functional regulatory elements (REs) affect transcriptional activity by altering the binding affinity or expression levels of TFs. Indeed, many noncoding variants associated with cardiovascular disease have been found to alter the binding affinities or probabilities of key cardiac TFs, including NKX2.5 (Benaglio et al., 2019), TBX5 (Gacita et al., 2021), ETS TF family members (Jindal et al., 2023), and MEF2 (Tan et al., 2020). TFs possess DNA-binding domains that recognize specific short DNA sequences, known as motifs. Within a motif, certain nucleotides are flexible, allowing the TF to bind regardless of the nucleotide. On the other hand, other nucleotides are highly conserved and essential for the binding affinity of the TF to the motif. Noncoding variants that alter a conserved motif nucleotide can impact the transcriptional activities through altering TF binding affinity or creating novel TF-binding sites (Deplancke et al., 2016; Lim et al., 2024). Depending on the function of TFs binding to the affected RE (activator vs. repressor), this disruption may lead to an increase or decrease in the expression of target genes. In cases where the TF of interest does not directly bind to the affected RE but relies on a co-factor within the transcriptional complex, the expression levels of the TF may become dysregulated (Liu et al., 2015). Notably, noncoding variants may introduce new binding sites for other TFs that do not typically bind to the region, resulting in alternative transcriptional programs. Hence, elucidating the mechanism by which a RE influences transcription often requires integrated and comprehensive investigation of gene expression, TF binding, histone modifications (e.g., H3K27ac), chromatin accessibility, chromatin conformation, and promoter activity.

Noncoding variants can also alter the 3D conformation of chromatin by disrupting pre-existing topologically associated domains (TADs), which are regions of the genome that come into physical contact with each other through spatially confined chromatin loops commonly defined by CTCF-binding sites. These chromatin loops bring REs into proximity with their target genes, facilitating transcriptional modulation (Grubert et al., 2020). Regions important for chromatin looping are often enriched with disease-associated noncoding variants and expression quantitative trait loci (eQTLs) (Bannister and Kouzarides, 2011; Lupiáñez et al., 2015). Disruption of these regions has been reported to drive epigenomic reprogramming in cardiomyopathy and heart failure (Feng et al., 2022), highlighting their critical role in gene regulation.

Noncoding variants located near exon-intronic junctions can influence the specific transcripts expressed for a gene through alternative or aberrant splicing, potentially leading to dysfunctional levels of the appropriate transcript or the production of an inappropriate splice variant, as seen with *MYBPC3* variants linked to hypertrophic cardiomyopathy (Lopes et al., 2020). Meanwhile, noncoding variants near the 5′ and 3′ untranslated regions can affect mRNA stability and processing by interfering with post-transcriptional processes such as polyadenylation and ribosome binding and assembly (Fu et al., 2024; Khan et al., 2022).

## Methodologies used in dissecting mechanisms of noncoding variants

Almost 98% of the genome comprises noncoding DNA (Lander, 2011), much of which remains poorly understood and requires thorough investigation using various assays. GWASs typically highlight several variants within an implicated locus, but which among these is the causal variant that drives the majority of disease risk is often difficult to establish. Other studies have reviewed approaches to identify the causal variant, from fine mapping to linkage disequilibrium (Stankey and Lee, 2023). Here, we focus on the methodologies used to study the impact of noncoding variants once a candidate causal variant has been established. Although these methods are discussed separately, it is the combination and integration of these methodologies that allows the comprehensive mechanistic investigation of noncoding variants and the identification of functional REs.

### Transcriptomics

Identifying differentially expressed genes between a control cell and a cell carrying a noncoding variant provides important information on genes and pathways affected by the presence of the noncoding variant. Since it is usually unknown which factors could be disrupted, an unbiased, high-throughput approach such as RNA sequencing is helpful. Since many REs show spatiotemporal and cell type-specific activity, single-cell RNA sequencing has emerged as a powerful technology to understand the cell or state-dependent effects of noncoding variants (Dou et al., 2024). In addition, RNA sequencing integrated with the genotype of individuals enables the generation of eQTL data, which provide a measure of how genetic variants affect gene expression at the population level (Nica and Dermitzakis, 2013). *cis*-eQTLs enable the study of gene expression in close proximity, while *trans*-eQTLs enable the study of distal genes that may be indirectly impacted by the noncoding variant. This becomes powerful in the context of identifying target genes of a noncoding variant, as it allows the study of gene expression changes in wild-type, heterozygous, and homozygous carriers of the variant. However, while transcriptomics data are critical, it is not sufficient by itself to study the mechanisms of noncoding variants and requires epigenomic profiling.

### TF binding and epigenomics

#### TF binding – Computational and biological assays

Computational tools to screen for both established and *de novo* motifs for TFs are typically employed first (Hecker et al., 2023), followed by validation of TF-binding sites by chromatin immunoprecipitation and qPCR (ChIP-qPCR) or ChIP sequencing (ChIP-seq) (Haring et al., 2007). ChIP-qPCR is employed to quantify TF binding at specific regions of DNA, while ChIP-seq provides a genome-wide analysis of TF-binding sites. More streamlined chromatin profiling techniques, such as CUT&Tag and CUT&RUN, have been developed to study TF-binding sites (Kaya-Okur et al., 2019). Preferential binding of a particular TF to a defined DNA sequence can be further interrogated through electrophoretic mobility shift assay (EMSA), which can be done *in vitro* and is better used for identifying the precise motif sequence as one can employ many variations of the DNA sequence at large scale (Hellman and Fried, 2007). These methods enable the investigation of whether a noncoding variant alters TF binding affinity and, if so, to what extent. For instance, to understand how an atrial fibrillation-associated variant may disrupt TF binding, one study leveraged a multitude of computational tools, such as motif prediction and intra-genomic replicate analyses, along with biological validation assays, including ChIP-qPCR and EMSA, which revealed a differential binding affinity of TFAP2a to the region harboring the variant, as discussed in more detail later in the review (Ye et al., 2016).

#### Chromatin accessibility and histone modification

Post-translational modifications in histone protein modifications play a crucial role in DNA packaging, organization, and regulation of chromatin structure, which in turn impact gene expression. Commonly investigated histone marks include acetylated histone 3 lysine 27 (H3K27ac), associated with accessible and actively transcribed chromatin; H3K4 mono-methylation (H3K4me1), associated with enhancer activity; H3K4me3, associated with promoter activity; and H3K27me3, associated with heterochromatin and gene repression (Bannister and Kouzarides, 2011). These data can inform the transcriptional activity of genes, particularly when combined with TF binding data. However, histone modifications usually spread over a relatively large stretch of DNA, which may be less useful for pinpointing smaller DNA REs that may be differentially accessible between cell types or experimental groups. To address this, genome-wide chromatin accessibility using transposase-accessible chromatin with sequencing (ATAC-seq) or cleavage under targeted accessible chromatin can be used to complement histone modification data (Grandi et al., 2022). Differentially accessible regions are then further analyzed bioinformatically to infer which TFs are bound to those differentially accessible REs (Buenrostro et al., 2015). In the context of noncoding variants, investigation of chromatin accessibility can provide insights into whether and how noncoding variants may alter the accessibility of important DNA regions.

#### Chromatin looping and architecture

While transcriptomics and chromatin accessibility profiling may identify candidate regulatory regions, the relationship between these regions and their target genes can be further elucidated through 3D chromatin conformation assays. To understand which genomic regions interact with each other and infer which gene promoters could be regulated by a specific RE (i.e., enhancer), 3D chromatin organization maps are generated using Hi-C, a high-throughput method to measure pairwise contacts between genomic loci (Grubert et al., 2020). Hi-C combines chromosome conformation capture (3C) technology, which enables the study of chromatin loops between non-adjacent genomic regions, with high-throughput sequencing (Dekker et al., 2002). Briefly, 3C involves cross-linking DNA to fix chromatin interactions in place, followed by digestion of the DNA and re-ligation of loose DNA ends. This process generates DNA fragments that contain pairs of genomic regions that are physically close in 3D space but not linearly adjacent. These interactions can then be analyzed to map the chromatin loops and architecture, providing insights into how REs communicate with gene promoters, and if those are disrupted by noncoding variants.

### Enhancer activity and specialized assays

Candidate REs, such as enhancers identified using epigenomics profiling, remain putative without functional validation of their *in vitro* activity. Luciferase assay is the most commonly used assay to measure the functional activity of a putative enhancer and whether variation in its sequence impacts its activity. By cloning the putative enhancer sequence into a luciferase reporter plasmid followed by delivery into cells, one can measure the increase in luciferase activity relative to baseline, representing the putative enhancer’s quantitative activity. By comparing enhancer sequences with or without the risk variant allele, the impact of the variant on enhancer activity can be directly assessed. The massively parallel reporter assay (MPRA) builds upon the concept of a luciferase assay and is used as a high-throughput assay for thousands of putative REs in parallel to identify those acting as enhancers. Briefly, an upstream RE plasmid library is generated, with each sequence uniquely barcoded. After introducing the library into cells, RNA extraction and gene expression analyses (e.g., RNA sequencing) enable large-scale measurement of reporter gene expression changes driven by each RE (Melnikov et al., 2012). These datasets can often be useful in first identifying functional enhancers followed by investigation of noncoding variants found to be within the enhancer sequences. This approach was recently used to identify cardiac chamber-specific enhancers in atrial and ventricular CMs harboring noncoding variants linked to electrocardiogram parameters (e.g., PR interval, QT interval) (Cao et al., 2023). While MPRA is a powerful tool, it relies on previous knowledge of RE sequences. In contrast, self-transcribing active regulatory region sequencing (STARR-seq) employs a similar approach to MPRA but takes a more unbiased way by using random genomic DNA fragments. Only those fragments containing functional enhancer sequences are transcribed. In this setup, DNA fragments containing potential enhancers are placed downstream of the reporter gene promoter, and if they are functional, they transcribe themselves, which enables sequencing of both the reporter gene and the sequence of functional REs (Arnold et al., 2013). This has been successfully leveraged in rat atrial CMs to identify atrial CM-specific enhancers and silencers within noncoding regions associated with atrial fibrillation, revealing some with allele-specific activity (van Ouwerkerk et al., 2019). Additionally, advances in clustered regularly interspaced short palindromic repeats (CRISPR) technology have provided valuable tools for epigenetic research. By targeting a catalytically inactive CRISPR protein to a RE and physically blocking any TFs or histone modifiers from binding, CRISPR interference (CRISPRi) allows for functional validation of putative regulatory regions *in vitro* and *in vivo* (Pacalin et al., 2024). Furthermore, CRISPR machinery can be engineered to include epigenetic modifiers, such as histone deacetylases and transcriptional repressors, to specifically modulate the transcriptional activity of a particular region (Kwon et al., 2017). CRISPRi-mediated epigenetic silencing by targeting REs of cardiac genes has been demonstrated to be feasible both *in vitro* and *in vivo* using an AAV9-based approach (Laurette et al., 2024).

## hiPSC models as a resource for generating epigenomics datasets

While all cells carry the same genome, cell type and state specificity are mediated by the spatiotemporal regulation of gene expression through REs and context-specific chromatin looping (Akerberg and Pu, 2020). To understand noncoding variants associated with cardiovascular disease, the species, cellular context, and developmental stage are critical factors. Given their ability to model cardiac disorders in a human context, with controlled differentiation and maturation stages, hiPSC-CMs have emerged as a powerful tool to study noncoding variants and gene regulation within the genome’s spatiotemporal framework. This has been particularly useful for generating epigenomics resources for the cardiac research community.

Gene expression in hiPSC-CMs has been reported to closely resemble that of heart tissue samples from the Genotype-Tissue Expression (GTEx) project. Moreover, eQTLs identified in hiPSC-CMs are most consistent with those found in GTEx left ventricular tissue, rendering hiPSC-CMs an effective model for studying the impact of noncoding variants on gene expression (Banovich et al., 2018). The similarity between hiPSC-CM and heart tissue gene expression has prompted the generation of large-scale hiPSC-CM-based eQTL datasets, such as a multi-ethnic eQTL dataset comprising hiPSC-CMs from 71 individuals (Lv et al., 2024), and another dataset featuring 2,500+ eQTLs from fetal-like hiPSC-derived cardiovascular precursor cells for developmental studies (D’Antonio et al., 2023). These resources are particularly powerful when integrated with adult heart tissue eQTL datasets (e.g., GTEx), enabling the dissection of cell type- and stage-specific eQTLs (Dababneh et al., 2024a; D’Antonio et al., 2023).

Mapping chromatin looping topography, using high-resolution Hi-C, has also been performed across the differentiation of hiPSCs to CMs, providing a comprehensive view of 3D chromatin architecture and epigenomic transitions from stem cells to CMs (Choy et al., 2018; Greenwald et al., 2019). Using these insights, one study has shown that the transcriptional transition during cardiac development is driven by genome reorganization, a critical mechanism by which enhancers interact with their target cardiac genes to facilitate differentiation (Choy et al., 2018). Another study, which mapped chromatin topography in hiPSCs and hiPSC-CMs from seven related, genotyped individuals, found that subtle changes in chromatin contact propensity at looped regions significantly impact gene regulation (Greenwald et al., 2019). This suggests that even minor genetic variation in REs involved in chromatin looping can result in considerable changes in gene expression, potentially leading to pathogenesis.

Other important epigenomic resources generated in hiPSC-CMs include chromatin accessibility maps using ATAC-seq, histone marks such as H3K27ac, H3K4me1, H3K4me3, and H3K27me3, and genome-wide binding profiles of cardiac TF, as highlighted in Table 1, which presents a thorough but not exhaustive list of datasets generated in hiPSC-based heart models.

**Table 1:** Select epigenomic and transcriptomic datasets generated in hiPSC-CMs

Category/target	Method	Cell type/stage	Accession #	Citation
**Histone modifications**				

H3K27ac	ChIP-seq	hiPSC-CM	GSE85628	Ang et al. (2016)
		hiPSC, hiPSC-CM	GSE125540	Greenwald et al. (2019)
		D0, D2, D5, D7, D15, D80 hESC-CM	GSE116862	Arvanitis et al. (2020)
	CUT&RUN	hiPSC-CM	GSE243902	Baudic et al. (2024)
H3K4me1	ChIP-seq	D0, D2, D5, D7, D15, D80 hESC-CM	GSE192365	Arvanitis et al. (2020)
	CUT&RUN	hiPSC-CM	GSE243902	Baudic et al. (2024)
H3K4me3	ChIP-seq	hiPSC-CM	GSE85628	Ang et al. (2016)
		hESC-CM	GSE35583	Paige et al. (2012)
		D0, D2, D5, D7, D15, D80 hESC-CM	GSE192365	Arvanitis et al. (2020)
	CUT&RUN	hiPSC-CM	GSE243902	Baudic et al. (2024)
H3K27me3	ChIP-seq	hiPSC-CM	GSE85628	Ang et al. (2016)
		hESC-CM	GSE35583	Paige et al. (2012)
		D0, D2, D5, D7, D15, D80 hESC-CM	GSE192365	Arvanitis et al. (2020)
H3K36me3	ChIP-seq	hiPSC-CM	GSE85628	Ang et al. (2016)
		hESC-CM	GSE35583	Paige et al. (2012)
		hiPSC-CPC	GSE159411	Gonzalez-Teran et al. (2022)

**Chromatin accessibility, looping, and promoter interactions**				

Chromatin accessibility	ATAC-seq	hiPSC-PC	GSE146044	Van Eif et al. (2020)
		hiPSC-PC	GSE85630	Ang et al. (2016)
		hiPSC-CM	GSE85330	Liu et al. (2017)
		hiPSC-CM	GSE243900	Baudic et al. (2024)
		hiPSC, hiPSC-CM	GSE133833	Benaglio et al. (2019)
		D0, D2, D5, D14 hESC-CM	GSE106689	Bertero et al. (2019)
		D0, D2, D5, D7, D15, D80 hESC-CM	GSE192365	Arvanitis et al. (2020)
	single-cell ATAC-seq	hiPSC-PC	N/A	Engel et al. (2023)
	single-nucleus ATAC-seq	D0, D2, D5, D7, D15, D25 hiPSC-CM (2D and 3D differentiation)	GSE245498	Holman et al. (2024)
CTCF binding	CUT&RUN	hiPSC-CM	GSE243902	Baudic et al. (2024)
Loops and promoter interactions	promoter capture Hi-C	hiPSC, hiPSC-CM	E-MTAB-6014	Montefiori et al. (2018)
		hESC-CM	GSE100720	Choy et al. (2018)
	Hi-C	hiPSC, hiPSC-CM	GSE125540	Greenwald et al. (2019)
		D0, D2, D5, D14 hESC-CM	GSE106687	Bertero et al. (2019)
		D0, D2, D5, D7, D15, D80 hESC-CM	GSE116862	Zhang et al. (2019b)

**Cardiac transcription factor binding sites**				

NKX2.5	ChIP-seq	hiPSC-CM	GSE133833	Benaglio et al. (2019)
		hESC-CM	GSE89443	Anderson et al. (2018)
		hiPSC-CPC	GSE159411	Gonzalez-Teran et al. (2022)
GATA4	ChIP-seq	hiPSC-CM	GSE85628	Ang et al. (2016)
		hiPSC-CPC	GSE159411	Gonzalez-Teran et al. (2022)
TBX5	ChIP-seq	hiPSC-CM	GSE81585	Churko et al. (2018)
		hiPSC-CM	GSE85628	Ang et al. (2016)
		hiPSC-CPC	GSE159411	Gonzalez-Teran et al. (2022)
HEY2		hiPSC-CM	GSE81585	Churko et al. (2018)
NR2F2	ChIP-seq	hiPSC-CM	GSE81585	Churko et al. (2018)
MEIS1	ChIP-seq	hiPSC-CPC	GSE159411	Gonzalez-Teran et al. (2022)
ISL1	ChIP-seq	hiPSC-CPC	GSE159411	Gonzalez-Teran et al. (2022)
GLYR1	ChIP-seq	hiPSC-CPC	GSE159411	Gonzalez-Teran et al. (2022)
MED1	ChIP-seq	hiPSC-CM	GSE85628	Ang et al. (2016)

**Transcriptomics**				

Gene expression	bulk RNA-seq	hiPSC-CM	GSE151279	Feyen et al. (2020)
		hiPSC-aCM, hiPSC-vCM	GSE111007	Cyganek et al. (2018)
		D0, D2, D5, D14 hESC-CM	GSE106688	Bertero et al. (2019)
	single-cell RNA-seq	D0, D5, D14, D25 hiPSC-CM	GSE81585/syn7818379	Churko et al. (2018)
		D0, D2, D5, D15, D30 hiPSC-CM	E-MTAB-6268	Friedman et al. (2018)
		hiPSC-PC	N/A	Engel et al. (2023)
		D0, D4, D5, D6, D10, D19 hiPSC-PC	GSE189782	Wiesinger et al. (2022)
		D0, D2, D5, D7, D15, D25 hiPSC-CM (2D and 3D differentiation)	CIRM CESCG: chiCardiomyocyte1	Holman et al. (2024)
eQTL	bulk RNA-seq + WGS	hiPSC-CPC	https://doi.org/10.6084/m9.figshare.c.5594121	D’Antonio et al. (2023)
	bulk RNA-seq + genotype	hiPSC-CM	https://guerratylab.org/ipsc/	Lv et al. (2024)

CM, cardiomyocyte; PC, pacemaker cell; CPC, cardiac progenitor cell; aCM, atrial cardiomyocyte; vCM, ventricular cardiomyocyte.

## Characterization of noncoding variants in hiPSC-CMs

**Figure 2:**
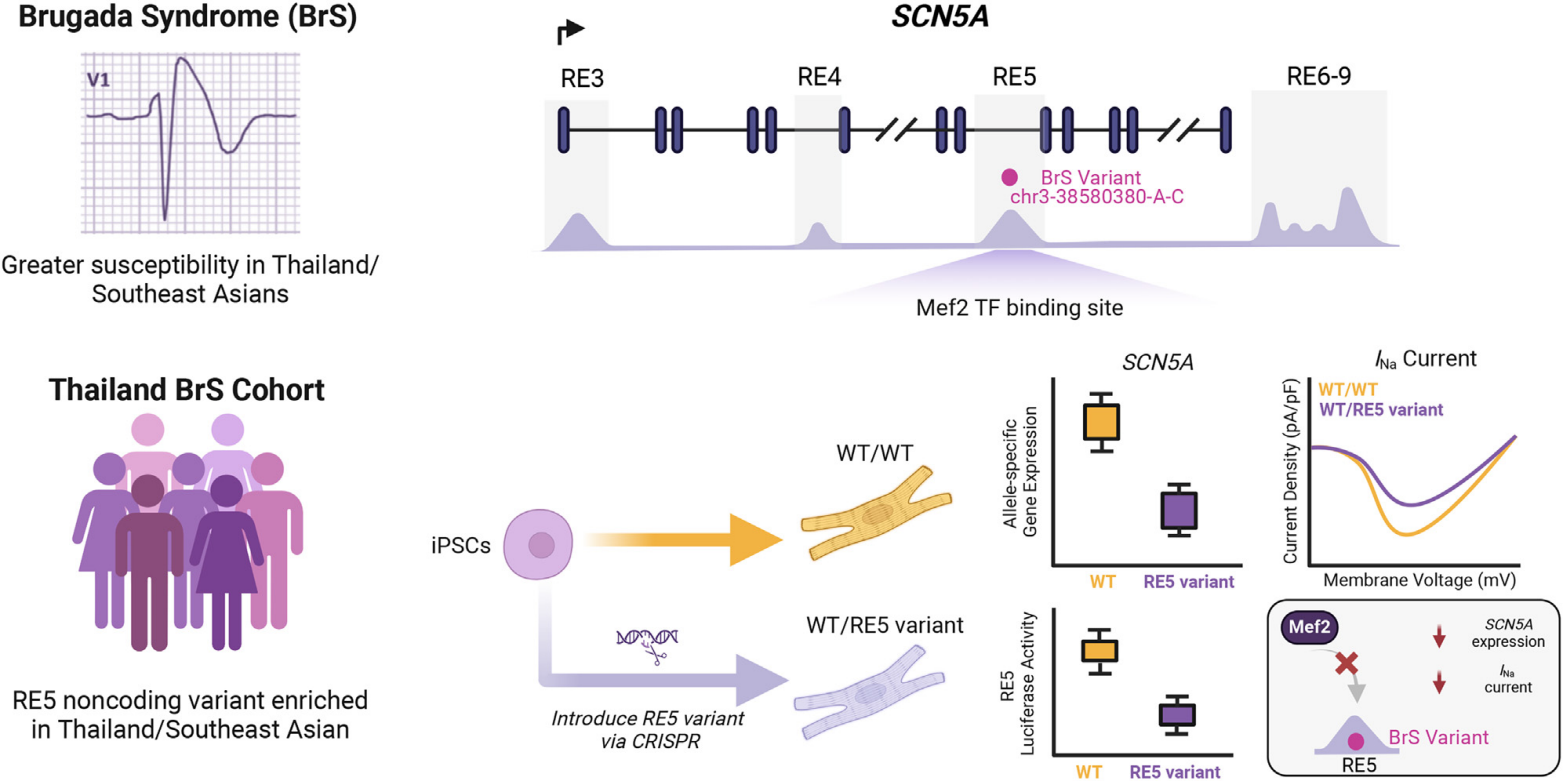
*SCN5A* noncoding variant underlying genetic susceptibility to Brugada syndrome in Southeast Asians A graphical summary of the study by Walsh et al. (2024) demonstrating the molecular mechanism of an intronic variant in RE5 of the *SCN5A* gene, which is particularly enriched in the Thailand/Southeast Asian BrS population. The illustrations are based on the data from Walsh et al. (2024). BrS, Brugada syndrome. Created with BioRender.

The use of hiPSC-CMs to dissect mechanisms of noncoding variants has rapidly increased given the conferred advantages of a human-based cellular model. In this section, we highlight key studies that have leveraged hiPSC-CMs to identify and elucidate the mechanisms underlying noncoding variants associated with cardiac development, arrhythmia, cardiomyopathy, and cardiotoxicity. The noncoding variants discussed are summarized in Table 2. To demonstrate the path from clinical discovery to mechanistic dissection, an example of a Brugada syndrome (BrS) noncoding variant study is portrayed in Figure 2.

**Table 2:** Summary of noncoding variants investigated in hiPSC-CMs

Phenotype	Variant ID	Genomic context	Mechanism	Citation
**Cardiac development**				

Congenital heart disease	N/A	promoter loop of *ADAMTS6*	• increased *ADAMTS6* expression • created a novel binding site for the TF serum response factor (SRF)	Xiao et al. (2024)
Atrial fibrillation and PR interval	rs3807989	functional RE bound by NKX2.5	• increased *SSBP3* expression • altered NKX2.5 binding	Benaglio et al. (2019)
P-wave duration	rs590041	functional RE bound by NKX2.5	• decreased *CAV1/2* expression • altered NKX2.5 binding	Benaglio et al. (2019)

**Arrhythmia**				

Atrial fibrillation	rs2595104	RE within intronic region of *PITX2a/b*	• decreased *PITX2c* expression and RE activity • reduced binding of TFAP2a	Ye et al. (2016)
	N/A (1.5 Mb deletion)	gene desert upstream of *PITX2*	• RE maintains a TAD housing *PITX2*; deletion leads to 3D chromatin reorganization • reduced *PITX2* expression in hiPSC-vCMs and increased *PITX2* expression in hiPSC-PCs	Baudic et al. (2024)
	rs12931021	RE within intronic region of *ZFHX3*	• decreased RE activity and *ZFHX3* expression	Jameson et al. (2023)
Brugada syndrome	GRCh38: chr3-38580380-A-C	RE within intronic region of *SCN5A*	• decreased *SCN5A* expression and reduced *I*_Na_ current • reduced RE activity • disruption of predicted Mef2-binding site and predicted gain of Gfi1B binding	Walsh et al. (2024)

**Cardiomyopathy**				

Hypertrophic cardiomyopathy	*MYBPC3*: c.1090 + 453C>T, c.1224-52G>A, c.1928-569G>T	intronic splice sites	• aberrant splicing of *MYBPC3*	Holliday et al. (2021)
Dilated cardiomyopathy	rs875908	RE upstream of *MYH7*	• RE important for transcriptional switch from *MYH6* to *MYH7* during cardiac development; deletion of RE led to decreased *MYH7* and increased *MYH6* expression • variant disrupts a predicted TBX5-binding site	Gacita et al. (2021)

**Cardiotoxicity**				

Anthracycline-induced heart failure	rs28714259	RE within intergenic region on Chr15	• disrupted glucocorticoid receptor binding • reduced RE activity • worsened contractile function and diminished upregulation of cardioprotective pathways	Wu et al. (2022)
	rs11140490	splice site of the first exon of *SLC28A3-AS1*	• cardioprotective mechanism likely via regulation of *SLC28A3-AS1* expression • predicted to alter the binding sites of 43 regulatory features	Magdy et al. (2022)

### Cardiac development and congenital heart disease

#### Cardiac TFs and cardiac development

Congenital heart disease (CHD) is present in 1% of births annually, often requiring urgent medical care and long-term follow-up (Zimmerman et al., 2020). There are thousands of noncoding variants associated with CHD; however, understanding which ones are likely to impact important regulatory regions on a large scale is challenging. In this landmark study, the authors developed a lentiviral MPRA (lentiMPRA) in hiPSC-CMs to examine 6,500+ *de novo* noncoding variants identified through whole-genome sequencing of 750 CHD trios (Xiao et al., 2024). Using this approach, 403 were found to affect cardiac RE activity, 10 of which were thoroughly investigated by genetically introducing them into hiPSC-CMs, including the identification of target genes and variant-mediated disruption of TF binding. For instance, a noncoding variant in the promoter loop of *ADAMTS6*, a gene previously linked to embryonic heart defects in mice (Prins et al., 2018), resulted in increased *ADAMTS6* expression in three independent homozygous hiPSC-CM lines and created a novel binding site for the TF serum response factor. Importantly, the study utilized the lentiMPRA dataset to develop a predictive regression model, termed EpiCard, to prioritize future noncoding variants, which was validated and found to be effective in an independent CHD cohort (Xiao et al., 2024).

NKX2-5 is a key TF in cardiac development, and its dysfunction can lead to congenital heart malformations and arrhythmias (Pashmforoush et al., 2004). It is hypothesized that many of the noncoding variants associated with cardiac disorders likely alter binding sites of master cardiac development TFs such as NKX2-5. To explore this hypothesis, one study combined genomic, transcriptomic, epigenomic, and NKX2-5 binding datasets from seven hiPSC-CM lines (Benaglio et al., 2019). This study identified approximately 2,000 single-nucleotide variants predicted to have an allele-specific impact on NKX2-5 binding, many of which were associated with electrocardiogram traits. Importantly, NKX2-5 binding was generally found to affect H3K27ac peaks at enhancers in hiPSC-CMs but not hiPSCs, and the identified noncoding variants showed significant eQTLs almost exclusively in cardiac tissue, suggesting that allele-specific NKX2-5 binding specifically alters cardiac-specific REs. Two prioritized noncoding variants, rs3807989 and rs590041, were experimentally validated in hiPSC-CMs using EMSA, luciferase assay, and CRISPRi. rs3807989 is associated with atrial fibrillation (AF) and the PR interval electrocardiogram trait (Ellinor et al., 2012; Pfeufer et al., 2010), while rs590041 is associated with P-wave duration (Verweij et al., 2020). These variants were shown to reside within functional REs typically bound by NKX2-5, modulating the expression of *SSBP3* and *CAV1/2*, respectively. Interestingly, rs3807989 increased the gene expression of *SSBP3*, while rs590041 decreased *CAV1/2* expression, indicating that NKX2-5 can play both activating and repressive roles at different REs. Future studies should implement similar approaches using hiPSC-CM data to investigate other key cardiac TF, such as TBX5 and GATA4, which may unveil more key REs commonly affected in congenital cardiac disorders.

#### Sinoatrial node development and function

The sinoatrial node (SAN) comprises specialized pacemaker cells that control the heart’s beating rate. Proper SAN development is critical for maintaining a normal heart rhythm. However, studying human pacemaker cell development in the SAN has been challenging due to the limited number of pacemaker cells with the complexity of their subtypes. In response to these challenges, the authors developed a hiPSC-based SAN differentiation protocol that yields large numbers of pacemaker cells from different SAN regions, including SAN head, SAN tail, and transition zone (Engel et al., 2023). Each of these pacemaker cell subtypes were analyzed for their molecular signatures using single-cell RNA and ATAC-seq. By dissecting the epigenetic and transcriptional determinants of each pacemaker cell subtype, the authors identified REs unique to each subtype. Notably, SAN tail and transitional REs were enriched for variants associated with resting heart rate, while SAN head REs were enriched for variants linked to heart rate recovery after exercise, supporting the notion of functional compartmentalization within the SAN.

In a separate study leveraging chromatin accessibility data from hiPSC-derived pacemaker-like cells, another group identified a key RE near the gene *MED13L*, which harbors SNPs associated with heart rate response after exercise (Van Eif et al., 2020). Using a mouse model, the authors found that this region is critical for *Tbx3* expression in pacemaker cells, with SNPs in this RE likely leading to aberrant *TBX3* expression in human nodal cells.

### Arrhythmia

#### AF

AF is the most common cardiac arrhythmia, affecting over 5% of the population over the age of 60 (Kornej et al., 2020). Interestingly, GWASs have revealed a strong genetic underpinning to AF susceptibility, with most variants located within noncoding regions (Ellinor et al., 2012). The 4q25 locus, harboring *PITX2*, has the strongest association with AF. *PITX2* is important for left-right asymmetry during cardiac development and is also implicated in AF risk (Hill et al., 2019; Steimle et al., 2022). However, the molecular mechanisms by which noncoding variants in this region contribute to AF risk remain poorly understood. Using hiPSC-CMs, one group demonstrated that the rs2595104 variant lies within an important RE in the intronic region of *PITX2a/b*, which modulates the expression of *PITX2c*, whose promoter is downstream of this RE. This finding was consistent with zebrafish reporter assays. Using ChIP-qPCR and EMSA in hiPSC-CMs, the authors uncovered the mechanism by which this variant affects PITX2c through the reduced binding of TFAP2a to the risk allele, relative to the non-risk allele. This study suggests that AF risk within this locus may be mediated through altered regulation of *PITX2* expression due to variants that disrupt TF binding at critical REs (Ye et al., 2016).

Variants disrupting the 3D chromatin organization, particularly in TADs and loops within the 4q25 locus, can also contribute to arrhythmias. One study examined seven families with an overlapping 1.5 Mb deletion in 4q25, who presented with a novel arrhythmia syndrome, including sinus node dysfunction, ventricular repolarization abnormalities, and structural defects. The deletion was found to be within a gene desert upstream of *PITX2*. To investigate the molecular mechanisms of this intergenic deletion, the authors surveyed chromatin accessibility, histone marks (H3K27ac, H3K4me1, H3K4me3), and CTCF binding in wild-type hiPSC-CMs, which revealed a candidate regulatory region within the deletion range that marked a TAD housing *PITX2* as the only coding gene. Deletion of this regulatory region in hiPSC-CMs containing the homozygous deletion led to profound reorganization of the 3D chromatin structure at the 4q25 locus. Given the differential role *PITX2* plays in atrial and ventricular cells, the authors then differentiated the genome-edited hiPSCs into ventricular and pacemaker-like CMs and measured *PITX2* expression. This revealed reduced expression of *PITX2* in ventricular-like cells and increased *PITX2* expression in pacemaker-like cells, mirroring findings in mouse hearts with the same deletion. Thus, by leveraging hiPSC-CMs to study human CM 3D conformation, this study uncovered the molecular mechanisms of a newly described cardiac syndrome resulting from the chromatin reorganization of the 4q25 locus identified in these families (Baudic et al., 2024).

Other AF-associated loci have also been investigated using hiPSC-CMs. For instance, another study identified a key RE found within an intron of *ZFHX3* at the 16q22.3 locus (Jameson et al., 2023). In mice, Zfhx3 plays an important role in atrial function, and its deletion leads to atrial dysfunction and arrhythmia. The identified RE was shown to be important for *ZFHX3* expression, with its deletion resulting in reduced expression. Interestingly, a variant within this RE, rs12931021, was found to reduce the RE activity and *ZFHX3* expression in homozygous carriers of the risk allele (AA) compared to the protective allele (CC), suggesting that altered *ZFHX3* expression through noncoding variants in this region confers AF risk.

#### BrS

Noncoding variants also contribute to inherited arrhythmias with earlier onset and greater risk of sudden death such as BrS. BrS is an inherited arrhythmia disorder that can cause sudden death in young adults and is primarily caused by pathogenic variants in the *SCN5A* gene, which encodes the *I*_Na_ current mediated by Na_v_1.5, leading to a loss of function with reduced *I*_Na_ current. Its prevalence is particularly higher in Southeast Asia, but the underlying ancestry-specific molecular factors are unclear (Makarawate et al., 2020). Genome sequencing in Southeast Asian patients with BrS and controls revealed a rare non-coding enhancer variant in the intronic region of the *SCN5A* gene, predicted to disrupt a MEF2-binding site. The introduction of this variant into hiPSC-CMs resulted in a significant reduction in *SCN5A* expression with a 30% decrease in *I*_Na_ density compared to isogenic controls. This finding provides causal evidence for the role of this variant in BrS and highlights ancestry-specific mechanisms underlying BrS disease susceptibility (Walsh et al., 2024). A graphical summary is shown in Figure 2.

### Cardiomyopathy

Hypertrophic cardiomyopathy (HCM) is the most common known cause of sudden cardiac arrest in young adults and athletes, most frequently resulting from pathogenic variants in *MYBPC3* (Lopes et al., 2024). Alternative splicing of *MYBPC3* is a known mechanism by which variants in this gene lead to HCM (Lopes et al., 2020), and several studies have modeled this mechanism in hiPSC-CMs (Dababneh et al., 2024b; Prondzynski et al., 2017). However, clinically confirming intronic splice-altering variants in *MYBPC3* via transcriptome sequencing is challenging, as it requires patient tissue expressing sufficient levels of disease-relevant transcripts, which are scarce and require invasive procedures. A recent study demonstrated the utility of hiPSC-CMs as a model to validate splice-altering variants and develop tailored treatments. The authors used HCM patient-derived hiPSC-CMs to confirm aberrant splicing in two patients with known *MYBPC3* splice-gain variants (c.1090 + 453C>T and c.1224-52G>A). Notably, this approach resolved a previously unclear genetic cause of HCM, which was found to be the result of diverse cryptic exon splicing caused by an *MYBPC3* variant (c.1928-569G>T) and then validated in cardiac tissue from an affected sibling. To test whether normal splicing could be restored, the authors used an antisense oligonucleotide treatment in patient-derived hiPSC-CMs, which completely inhibited aberrant exon splicing, suggesting a potential tailored treatment for these patients (Holliday et al., 2021).

*MYH7* and *LMNA* are also well-established cardiomyopathy genes, with several known pathogenic coding variants associated with HCM and dilated cardiomyopathy (Hershberger et al., 2018). However, the role of noncoding variants affecting these genes in cardiomyopathy remains unclear. By combining epigenetic and TF binding signatures from human heart samples and hiPSC-CMs, including promoter-enhancer interaction maps, the authors identified several important enhancers for *LMNA* and *MYH7*. One enhancer, located ∼2 kb upstream of the *MYH7* promoter, was essential for the transcriptional switch from *MYH6* to *MYH7* during cardiac development. *MYH7* is the dominant form of myosin heavy chain in the human heart, which demonstrates slower but more forceful contractile dynamics compared to *MYH6* (Solaro, 2010). Deletion of this enhancer in hiPSC-CMs resulted in a dramatic reduction in *MYH7* expression, accompanied by a dose-dependent increase in *MYH6* expression and faster contraction in engineered heart tissue derived from hiPSC-CMs. Using a computational pipeline, the authors identified the noncoding variant rs875908 within this enhancer, which was predicted to disrupt a previously identified TBX5-binding site. Patients carrying the rs875908 risk allele were found to have a greater risk of severe dilated cardiomyopathy, corroborating the importance of this noncoding variant as a potential cardiomyopathy biomarker. Additionally, this approach enabled the discovery of a *MYH6/7* enhancer likely required for the chromatin reorganization underlying cardiac development, which may be involved in other diseases exhibiting aberrant contractile function (Gacita et al., 2021).

### Cardiotoxicity

Anthracyclines such as doxorubicin, which are used to treat cancers, are known to increase the risk of heart failure, with prominent underlying genetic susceptibility (Aminkeng et al., 2015; Huang et al., 2022). The rs28714259 variant is associated with an increased risk of anthracycline-induced heart failure, but the mechanism is unclear (Schneider et al., 2017). Using hiPSC-CMs harboring rs28714259, a study revealed that this variant increases the risk of anthracycline-induced heart failure by disrupting glucocorticoid receptor signaling, which is activated by dexamethasone pretreatment. Dexamethasone provides cardioprotective effects against cardiotoxicity, but rs28714259 significantly disrupts the binding of the glucocorticoid receptor to this regulatory region and reduces enhancer activity compared to the major allele, as demonstrated by EMSA, ChIP-qPCR, and luciferase assay experiments in hiPSC-CMs. Additionally, hiPSC-CMs carrying rs28714259 exposed to doxorubicin showed worsened contractile function, an increased beating rate (marker of cardiotoxicity), and diminished upregulation of cardioprotective gene pathways following dexamethasone pretreatment. These findings suggest that rs28714259 could serve as a biomarker to predict the risk of anthracycline-induced heart failure (Wu et al., 2022).

Noncoding variants have also been identified to be protective against anthracycline-induced cardiotoxicity, such as the intronic variant rs885004 in the gene *SLC28A3* (solute carrier family 28 member 3) (Visscher et al., 2012). To identify the lead variant within this locus, the study performed Nanopore-based fine-mapping and base editing, which led to the identification of the lead noncoding variant within this region, rs11140490. This variant was found to protect against cardiotoxicity by regulation of the antisense long noncoding RNA *SLC28A3-AS1*, which influences the expression of doxorubicin-related genes. Notably, given the critical role *SLC28A3* plays in cardiotoxicity, the authors performed a high-throughput drug screening in hiPSC-CMs, followed by *in vivo* validation in mice. This led to the identification of desipramine as an effective competitive inhibitor of SLC, providing protection against cardiotoxicity (Magdy et al., 2022). Altogether, this work highlights the utility of hiPSC-CMs in all stages of noncoding variant analysis, from mechanistic discovery to therapeutic development, enabling rapid translation of therapies that may benefit patients.

## Future of hiPSC-CMs as a model to dissect noncoding genetic variation

**Figure 3:**
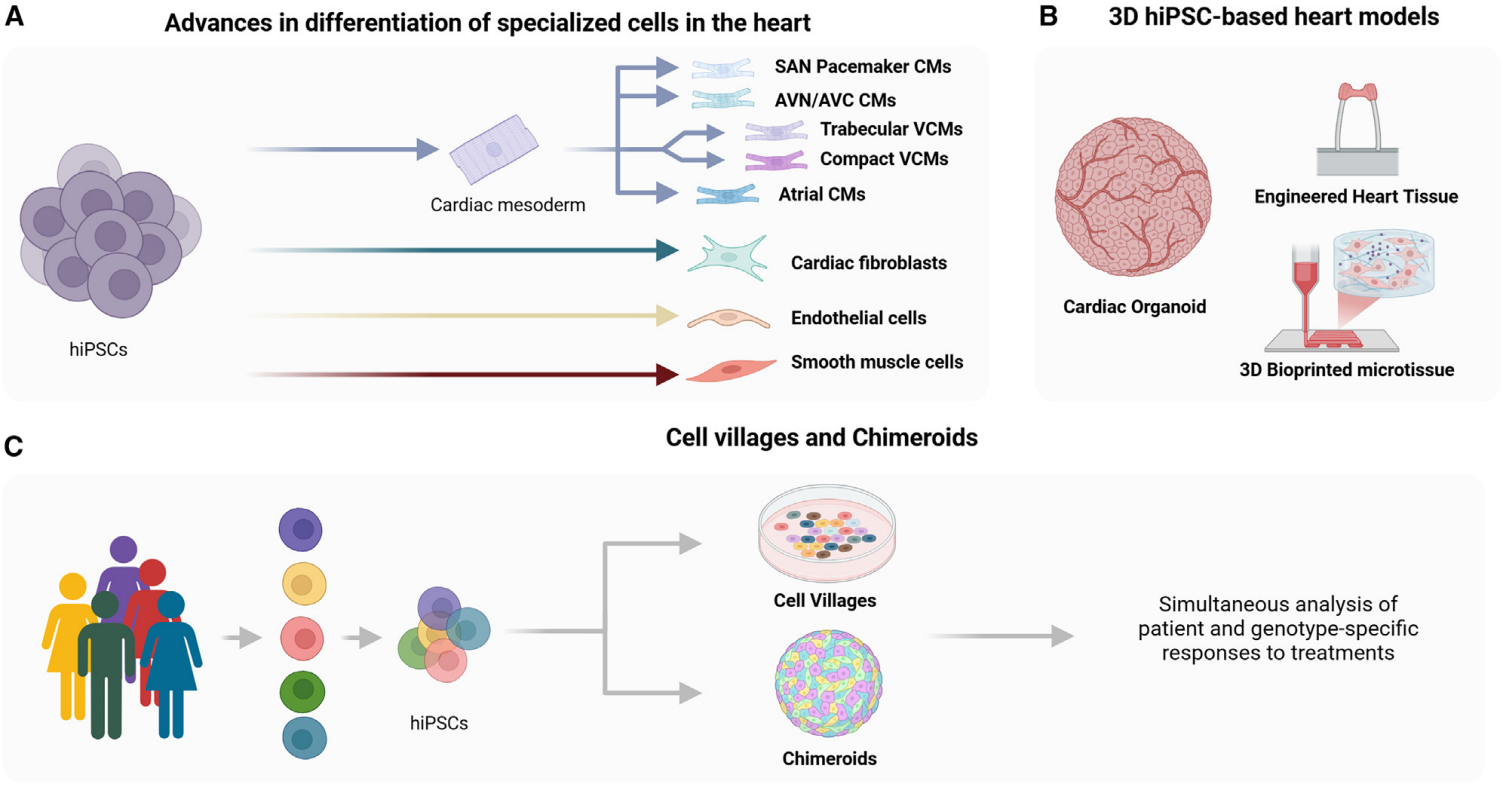
Advances in cardiac hiPSC-based models (A) hiPSCs can be differentiated into several cell types native to the human heart, including cardiomyocyte subtypes (atrial, ventricular, compact, trabecular, sinoatrial node, and atrioventricular node), endothelial cells, cardiac fibroblasts, and smooth muscle cells. (B) 3D hiPSC-based heart models including cardiac organoids, engineered heart tissue, and 3D bioprinted tissue. (C) Modeling human variation through advances such as cell villages and chimeroids enables simultaneous dissection of several individuals’ cells and their respective responses to treatments. SAN, sinoatrial node; AVN, atrioventricular node; AVC, atrioventricular canal; VCM, ventricular cardiomyocyte. Created with BioRender.

Rapid advances in the hiPSC field are enabling modeling of complex phenotypes in more cell type-, cell stage-, and 3D geometry-specific fashions. Here, we briefly discuss advances in differentiation protocols to generate specific hiPSC-derived cell types, improved maturation methods, hiPSC-based 3D tissues and organoids, and the emerging concepts of cell villages and chimeroids to study noncoding human variation on a large scale (Figure 3). We have previously discussed in detail the various methods utilized in the maturation of 2D hiPSC-CM models, which are not detailed here (Dababneh et al., 2024b; Hamledari et al., 2022).

### hiPSC models in the context of preclinical models

Our basic understanding of human gene regulation has largely been derived from animal models, including mouse, zebrafish, rat, *Drosophila*, and *C. elegans*, all of which have been instrumental in advancing this field. The subsequent development of hiPSCs has undoubtedly revolutionized human disease modeling, particularly in the cardiac field, given the difficulty of obtaining cardiac biopsies and culturing primary human CMs. The use of hiPSCs to investigate noncoding variants associated with cardiovascular diseases provides several advantages. There are well-established differences in electro-contractile coupling and cardiac ion channel profiles across species (Milani-Nejad and Janssen, 2014). In addition, while gene regulation may be conserved in certain genomic regions, other regions may display species-specific regulatory mechanisms (Lin et al., 2014). Thus, hiPSC-based models offer a robust platform to study cardiac function and dissect the function of REs in a species- and patient-specific manner. Additionally, parallel advancements in genome editing technology have poised hiPSCs as an attractive model to study rare genetic disorders by introducing variants and generating isogenic controls. This is particularly transformative for studying variants with low frequencies in the population (Hockemeyer and Jaenisch, 2016). hiPSC models are also highly advantageous for high-throughput screens (Edginton-White et al., 2023; Xiao et al., 2024), given that they serve as a theoretically unlimited source of human CMs, which can be generated on a large scale using bioreactors (Prondzynski et al., 2024).

However, significant challenges remain in the use of hiPSC models, including the immaturity in several cardiac parameters compared to primary adult cells, a relative lack of 3D tissue complexity compared to *in vivo* models, and the absence of interorgan communication and physiological feedback mechanisms, which could provide critical developmental and epigenetic cues (Clancy et al., 2024). Important advances have been made in addressing some of these limitations, which are discussed further. Since every model has its limitations, the combination of hiPSC models with animal models, which we highlight in this review where applicable, provides excellent synergy in uncovering the roles of noncoding variants at cellular, tissue, and organismal levels.

### Advances in differentiation, complexity, and maturity of cardiac hiPSC models

#### Differentiation into specialized cell types

While CMs are the contractile cells of the heart, the majority of heart cells are of other cell types such as fibroblasts, endothelial cells (ECs), and resident immune cells, all of which largely influence one another. Tremendous progress has been made in generating hiPSC-derived fibroblasts (Zhang et al., 2019a), ECs (Lian et al., 2014; Liu et al., 2023), and smooth muscle cells (SMCs) (Kwong et al., 2019), which can be co-cultured or bioprinted together to study cellular crosstalk and its impact on CM function and to explore the impact of the same noncoding variant on each cell type in isolation and together. Indeed, these advancements have already been leveraged to understand the noncoding genome in non-cardiac cells found within the human heart. One group utilized a multi-omics approach in hiPSC-derived SMCs to identify candidate enhancers harboring variants associated with blood pressure, peripheral arterial disease, and intracranial aneurysms (Liu et al., 2023). Another group focused on dissecting a large noncoding region on chromosome 8 associated with bicuspid aortic valve by studying endothelial-to-mesenchymal transition in hiPSC-derived ECs. Using this approach, the study demonstrated that this noncoding region modulates *GATA4* expression and that the rs117430032 variant disrupts *GATA4* expression dynamics likely by affecting TWIST1 binding in this region (Huang et al., 2023). Additionally, another group employed hiPSC-ECs and hiPSC-SMCs to study rs9349379, an intronic variant within the *PHACTR1* gene associated with five vascular diseases, showing that this variant regulates the expression of endothelin 1, a potent vasoconstrictor released into the bloodstream by ECs (Arvanitis et al., 2020).

Moreover, the cardiac conduction system is composed of unique and specialized cells with distinct functional properties and transcriptional programs, including the sinoatrial and atrioventricular node, *His* bundle, bundle branches, and *Purkinje* fibers (Oh et al., 2024). Important advances have been achieved in the ability to generate hiPSC-derived sinoatrial pacemaker cells (Protze et al., 2017; Wiesinger et al., 2022) and atrioventricular nodal cells (Li et al., 2024; Ye et al., 2024), in the past few years, enabling precise modeling of conduction disorders specific to these cell types, such as sick sinus syndrome and atrioventricular blocks, respectively. With a deeper understanding of the developmental pathways underlying the cardiac conduction system’s specialized cell types, hiPSCs may eventually be used to generate all of the conduction system’s subtypes, facilitating the study of noncoding variants and their unique mechanisms in each subtype.

#### Maturation and complex 3D models

Challenges in 2D cultures, such as those related to metabolic, mechanical, and electrical properties, can be addressed by generating hiPSC-based 3D cardiac tissue, which further improves cardiac properties and maturation (Correia et al., 2018). These 3D cardiac tissues can also be composed of both CMs and non-CMs. In one study, 3D-engineered heart tissue was created using hiPSC-CMs, SMCs, and cardiac fibroblasts, all sharing the same genomic background from the same individual. After a 3-week culture, the CMs showed an organized structure, enhanced conduction velocity and contractile force, and an increase in the formation of T-tubules and intercalated disc-like structures (Shadrin et al., 2017). Another study showed improved cardiac structure, electrophysiology, and metabolism when combining hiPSC-CMs with hiPSC-derived cardiac fibroblasts and ECs to generate 3D micro-tissues (Giacomelli et al., 2020).

Cardiac organoids (i.e., cardioids) are another 3D approach to improve CM maturation and more closely recapitulate the 3D properties of the heart (Buono et al., 2020; Li et al., 2018; Silva et al., 2021). Cardioids are hollow inside, which resembles the structure of the human heart chambers. Multi-cellular cardioids generated using CMs and non-CMs produce a better-aligned syncytium, tight cell-cell and cell-extracellular matrix connections, and improve electromechanical signal conduction (Giacomelli et al., 2020). Cardioids can also be vascularized, which allows for studying the impact of noncoding variants on the vasculature and their effects on CMs. One study showed that including matrix-secreting fibroblasts, ECs, and muscle cells within cardioids improved physiological stiffness and microvasculature (Giacomelli et al., 2017). Multi-chambered cardioids have also been generated, demonstrating compartment-specific structure and function (Schmidt et al., 2023).

### Cell villages and chimeroids

The cell village model involves co-culturing distinct hiPSC lines from different individuals in a shared environment to investigate various cell lines and genetic backgrounds simultaneously (Neavin et al., 2023). This concept enables studying genomic background-specific effects on gene expression or drug responses, through studying developmental stage and cell type-specific eQTLs more cost-effectively and on a larger scale. Interestingly, cell village studies have demonstrated that co-cultured hiPSC lines in a village environment retain their cell line-dependent genetic, epigenetic, and gene expression profiles (Cuomo et al., 2020; Jerber et al., 2021). While this approach to assess genomic variation in CMs has not been undertaken yet, one study showed the feasibility of differentiating distinct hiPSC lines in co-culture into CMs, although the number of unique hiPSC lines at the end of differentiation decreased significantly due to the varying growth rates among the lines (Neavin et al., 2023).

An adaptation of the cell village idea is chimeroids, which are organoids created by the aggregation of multiple distinct hiPSC lines. This model has been employed to study individual variations in response to neurotoxic triggers using single-cell RNA sequencing to dissect hiPSC-line-specific responses (Antón-Bolaños et al., 2024). This could be a powerful approach to investigate the impact of genetic background and noncoding variants on disease phenotype or drug responses, enabling large-scale phenotyping and pharmacogenomics studies on complex 3D structures such as the brain or the heart. However, the generation of cardiac chimeroids has not been reported to date. While these approaches hold great potential, the intrinsic heterogeneity of these models, along with batch-to-batch variations in differentiation, may necessitate additional tools to detect biologically meaningful signals effectively.

## Conclusion

Altogether, noncoding variants account for the majority of genetic associations with cardiovascular disease, and hiPSC-based cardiac models have emerged as powerful tools for dissecting the functional impact of noncoding genetic variants. These models enable the study of disease-relevant cell types in a controlled, patient-specific manner, bridging the gap between genetic association studies and molecular mechanisms. Ultimately, a better understanding of noncoding genetic variation and its impact on cardiac biology can pave the way for more precise diagnostic and therapeutic strategies targeting the genetic underpinnings of cardiovascular disease.

## Acknowledgments

This work was supported by the Heart and Stroke Foundation of Canada (HSFC) to G.F.T. S.F.D. is supported by the MD/PhD studentship (10.13039/501100005247University of British Columbia) and the Canada Graduate Scholarship—Doctoral Award (Canadian Institutes of Health Research, 193439). K.-H.K. is supported by the National New Investigator Award from the HSFC and the Early Researcher Award from the Government of Ontario, Canada. S.S.T. is supported by the Stem Cell Network Early Career Researcher Jump Start Awards Program (AWD-021244). V.J.-S. is supported by Daniel Bravo Andreu Private Foundation Award for research stays abroad and the Michael Smith Health Research BC Research Trainee award.

## Author contributions

S.F.D. and G.F.T. conceived the manuscript idea. S.F.D., H.B., and V.J.-S. wrote the initial draft of the manuscript. S.F.D., S.S.T., K.-H.K., and G.F.T. edited the manuscript. All authors approve the final version of the manuscript.

## Declaration of interests

The authors declare no competing interests.
